# Porcine cells restrict human cell proliferation via cellular competition in a human-porcine mesenchymal stem cells co-culture model

**DOI:** 10.3389/fcell.2026.1750289

**Published:** 2026-02-25

**Authors:** Xinyuan Fan, Xinglan An, Tong Zhang, Ziyi Li, Xiangpeng Dai, Xiaoling Zhang

**Affiliations:** 1 Key Laboratory of Organ Regeneration and Transplantation of Ministry of Education, First Hospital, Jilin University, Changchun, China; 2 National-Local Joint Engineering Laboratory of Animal Models for Human Disease, First Hospital, Jilin University, Changchun, China

**Keywords:** cell competition, human, mesenchymal stem cells, porcine, xenotransplantation

## Abstract

The xenotransplantation of human cells into porcine hosts holds immense potential in the fields of regenerative medicine and organ transplantation. However, the low survival rate of human-derived cells within porcine remains a critical bottleneck constraining the application of xenotransplantation. Whether porcine cells exert negative effect on human cell growth is not studied. Here, we established an *in vitro* direct co-culture model of human and porcine mesenchymal stem cells (hMSCs and pMSCs) to investigate the competitive relationship between human and porcine-derived cells. The results demonstrated that the proliferation capability of hMSCs in the co-culture system was significantly suppressed compared to those cultured in isolation. Moreover, an increasing number of pMSCs exhibited enhanced inhibition of hMSC proliferation. Notably, results from transwell assays and treatment with porcine-conditioned medium indicated that the inhibition of hMSCs by pMSCs was not mediated through soluble cytokines. To elucidate the underlying molecular mechanisms, RNA sequencing analysis was performed and the result revealed that direct co-culture significantly downregulated the expression of proliferation-related genes in hMSCs, including *CYP1B1*, *SLC7A11*, *TFAP2C*, and *PSAT1*. Concurrently, the co-culture paradigm disrupted endoplasmic reticulum function and multiple amino acid transport processes within hMSCs, while activating the NF-κB signaling pathway, thereby achieving negative regulation of hMSC proliferation. Collectively, our primary study characterized the competitive interactions between hMSCs and pMSCs and uncovered possible underlying mechanisms which provided new experimental foundations for improving human cell survival in porcine hosts to advance xenotransplantation application.

## 1 Introduction

Transplantation of human cells into porcine hosts exhibits immense potential in the fields of regenerative medicine and organ transplantation by generating physiologically functional humanized organs in pigs, establishing disease models, and evaluating the safety and efficacy of human cell therapies in porcine hosts (Wu et al., 2017). However, low human cell transplantation efficiency constrained the generation of humanized organs in pigs which can be clinically used in organ transplantation. Therefore, researchers tried to generate human cell lines with enhanced adaptability by overexpressing the anti-apoptotic gene *BCL2*, the pro-proliferative gene *MYCN*, or by mutating the tumor suppressor gene *TP53* in human cells (Maeng et al., 2021; Wang et al., 2023). Notably, the genetically modified cells not only survived and engrafted successfully in pig embryos with organogenesis defects but also contributed to the formation of functional humanized tissues, including endothelial tissue, skeletal muscle tissue, mid-stage renal structures, and cardiac muscle tissue (Das et al., 2020; Maeng et al., 2021; Wang et al., 2023; Mallapaty, 2025). These results demonstrate that targeted gene editing can effectively improve human cell survival in xenotransplantation settings and offer valuable insights for enhancing transplantation efficiency in future studies.

Importantly, the low survival rate of human cells in porcine hosts may be attributed to immune rejection and interspecies microenvironmental incompatibility. The porcine immune system identifies and attacks implanted human cells, leading to a complex immune rejection process. Moreover, the differences between human and porcine cellular microenvironments encompass three key aspects: interspecies cell competition, impaired intercellular signaling, and the potential risk of infection from porcine endogenous retroviruses (Schuurman et al., 2003; Niu et al., 2021; Zheng et al., 2021; Liu et al., 2023). Among these, cell competition—a widespread homeostatic regulatory mechanism in multicellular organisms—is regarded as a key determinant affecting interspecies cellular coexistence. Its primary role is to eliminate growth-compromised, functionally abnormal, or potentially malignant cells from tissues through selective removal (van Neerven and Vermeulen, 2022), thereby maintaining organismal health and homeostasis. This phenomenon was first described in studies of *Drosophila*, where cells from flies carrying a heterozygous mutation in the Minute gene (*Minute*+/−) survived and proliferated normally in isolation, exhibiting only mild phenotypic alterations such as reduced body size. However, they were recognized and selectively eliminated by apoptosis when placed adjacent to wild-type cells (Gines and Ripoll, 1975). This contact-dependent clearance provided the first evidence that cell competition acts as an adaptive mechanism for removing less-fit cells. Subsequent studies have demonstrated that not only genetically defective cells but also those with reduced proliferation rates, impaired damage-repair capacity, or metabolic disturbances can be eliminated by surrounding fitter cells via this process (Plusa et al., 2008). These findings offer an important theoretical basis for understanding the obstacles to interspecies cell coexistence.

Mesenchymal stem cells (MSCs) are a class of adult stem cells characterized by their self-renewal capacity, multipotent differentiation potential, and immunomodulatory functions, which hold significant value in the field of regenerative medicine (Han et al., 2019). As pivotal regulators of the tissue microenvironment, MSCs are widely distributed in the stroma of bone marrow, adipose tissue, and connective tissue. Elucidating the mechanisms by which MSCs exert their effects in cellular competition can reveal their crucial roles in various physiological and pathological processes, including tissue regeneration, stem cell transplantation and organismal aging (Marques-Reis and Moreno, 2021; Nowlan et al., 2021; Huang et al., 2023; Barcelona-Estaje et al., 2024; Khandekar and Ellis, 2024). Compared to pluripotent stem cells (PSCs), bone marrow-derived MSCs more closely approximate *in vivo* physiological conditions and possess distinct advantages, including low immunogenicity, reduced tumorigenic potential, and fewer ethical concerns (Adelipour et al., 2023). These features make MSCs an ideal model for both *in vitro* experimentation and *in vivo* transplantation studies.

Notably, human MSCs (hMSCs) and porcine MSCs (pMSCs) exhibit high similarity in core biological characteristics and functionalities. In terms of cell morphology, both cell types adhere to substrates and display a typical spindle shape, with hMSCs presenting a more elongated form compared to pMSCs. Regarding surface marker expression, both cell types demonstrate cross-species conservation. In terms of proliferation capacity, there are no significant differences between hMSCs and pMSCs in their *in vitro* growth potential (Noort et al., 2012). Concerning differentiation potential, both cell types possess stable tri-lineage differentiation capabilities towards osteogenic, adipogenic, and chondrogenic lineages. Immunologically, both hMSCs and pMSCs exhibit low immunogenicity and significant immunomodulatory activity, effectively suppressing phytohemagglutinin-induced T-cell proliferation (Comite et al., 2011). Based on these characteristics, this study selected hMSCs and pMSCs as research models to systematically describe the competitive interactions between them and elucidate the underlying molecular mechanisms governing this process. Ultimately, our work aims to provide new experimental foundations for improving human cell survival in porcine hosts and advancing the study of xenotransplantation.

## 2 Results

### 2.1 Isolation and characterization of pMSCs

**FIGURE 1:**
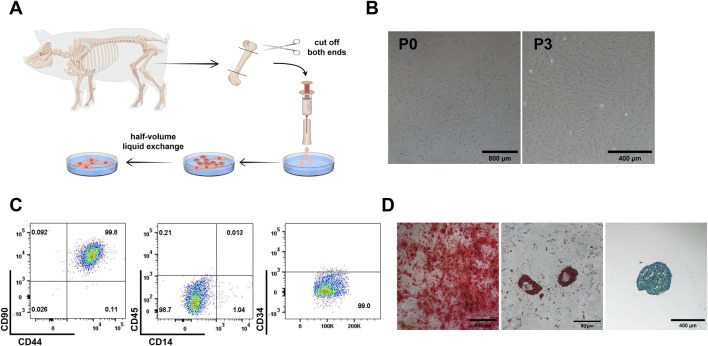
Extraction and characterization of pMSCs. **(A)** Schematic diagram of the pMSCs extraction process by Figdraw. **(B)** Representative images of the primary pMSCs. **(C)** Flow cytometric analysis of surface marker on pMSCs. **(D)** Identification of the multi-lineage differentiation potential of pMSCs.

pMSCs were isolated from the bone marrow of femurs and tibiae obtained under aseptic conditions from healthy piglets. Initially, primary (P0) pMSCs appeared scattered after adhesion. After 72 h, pMSCs proliferated to form distinct colony clusters. Upon passaging, the cells adopted a homogeneous, elongated, spindle-shaped morphology (Figures 1A,B). The isolated cells were positive for the mesenchymal stem cell markers CD90 and CD44 (both >98% positivity) and negative for the hematopoietic and immune markers CD4, CD14, CD19, CD34, CD45 and CD80, confirming a characteristic pMSC surface phenotype (Figure 1C; Supplementary Figures S1A–C). The multipotent differentiation potential was evaluated by inducing osteogenic, adipogenic, and chondrogenic lineages. After induction, the formation of calcium nodules was confirmed by Alizarin Red staining, lipid droplet accumulation was revealed by Oil Red O staining, and glycosaminoglycan deposition in the cartilage matrix was detected by Alcian Blue staining (Figure 1D). These results verify that pMSCs have the capability to differentiate into osteocytes, adipocytes, and chondrocytes. In summary, through morphological assessment, surface marker analysis, and multilineage differentiation assays, we successfully isolated and expanded highly pure and functional pMSCs.

### 2.2 pMSCs inhibit hMSCs proliferation by inducing apoptosis in the direct co-culture system

**FIGURE 2:**
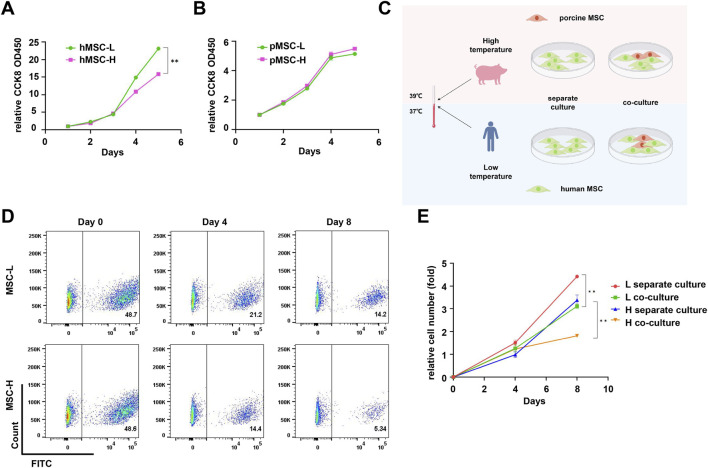
Direct co-culture of human and porcine MSCs. **(A)** The proliferation of hMSCs cultured at 37 °C and 39 °C. **(B)** The proliferation of pMSCs cultured at 37 °C and 39 °C. **(C)** Schematic diagram illustrating the direct co-culture system of human and porcine MSCs by Figdraw. **(D)** The percentage of GFP-labeled hMSCs under direct co-culture conditions at 37 °C and 39 °C (pMSCs: hMSCs = 1: 1). **(E)** The proliferation curve of hMSCs under direct co-culture conditions at 37 °C and 39 °C (pMSCs: hMSCs = 1: 1). Data are represented as mean ± standard error. *, *P* < 0.05; **, *P* < 0.01.

The efficacy of human cell xenotransplantation into porcine hosts may be limited by interspecies physiological differences, particularly with respect to body temperature and the competition among xenogeneic cells. We first investigated the impact of temperature using CCK-8 assays. The results indicated that the physiological temperature of pigs (39 °C) significantly inhibited the proliferation of hMSCs. In contrast, the proliferation of pMSCs was not significantly affected when comparing 37 °C–39 °C, underscoring their strong adaptability to human body temperature. These findings demonstrate that the porcine thermal environment exerts a substantial inhibitory effect on hMSCs (Figures 2A,B).

We then investigated the interactions between human and porcine cells by four experiments: hMSCs were cultured at 37 °C (L separate culture), hMSCs were cultured at 39 °C (H separate culture), hMSCs were co-cultured with pMSCs at 37 °C (L co-culture), and hMSCs were co-cultured with pMSCs at 39 °C (H co-culture). The proliferation of GFP-labeled hMSCs was evaluated using cell counting and flow cytometry (Figure 2C). Flow cytometric analysis revealed a time-dependent decline in the proportion of GFP-positive hMSCs during co-culture. Compared to the separate culture group, the proliferation rate of hMSCs was significantly reduced under co-culture conditions (Figures 2D,E). These results indicate that pMSCs could impair the survival and proliferation of hMSCs under co-culture conditions at both 37 °C and 39 °C, thereby influencing the overall efficiency of human cell xenotransplantation.

**FIGURE 3:**
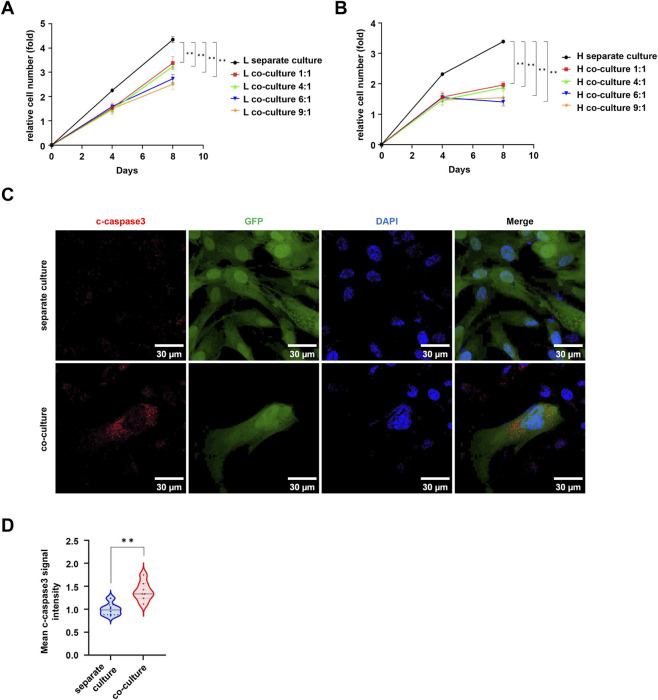
The effect of different proportions of pMSCs on the proliferation of hMSCs. **(A)** The effects of different inoculation ratios of pMSCs on the proliferation of hMSCs at 37 °C. **(B)** The effects of different inoculation ratios of pMSCs on the proliferation of hMSCs at 39 °C. **(C)** The expression of c-caspase3 protein in hMSCs within the direct co-culture system. **(D)** Statistical analysis of the mean fluorescence intensity of c-caspase3 protein. Data are represented as mean ± standard error. *, *P* < 0.05; **, *P* < 0.01.

Given that porcine cells significantly outnumber human cells during xenotransplantation of human cells into pigs, we examined the effect of pMSC concentration on hMSC proliferation through five experimental groups: hMSCs cultured alone and hMSCs co-cultured with pMSCs at cell number ratios of 1:1, 1:4, 1:6, and 1:9. The results indicated that higher pMSC ratios led to a stronger inhibition of hMSC proliferation, which was consistently observed at both 37 °C and 39 °C (Figures 3A,B). Notably, the higher temperature (39 °C) and co-culture exerted a synergistic inhibitory effect on hMSC proliferation. To investigate the underlying mechanism, immunofluorescence analysis was performed, and the results revealed a markedly increased expression of the apoptosis-related protein cleaved caspase-3 in GFP-labeled hMSCs under co-culture conditions compared to the control (Figures 3C,D), suggesting that pMSCs may inhibit proliferation by inducing apoptosis in hMSCs through undefined mechanisms.

### 2.3 The soluble factors secreted by pMSCs are not the primary agents inhibiting hMSC proliferation

**FIGURE 4:**
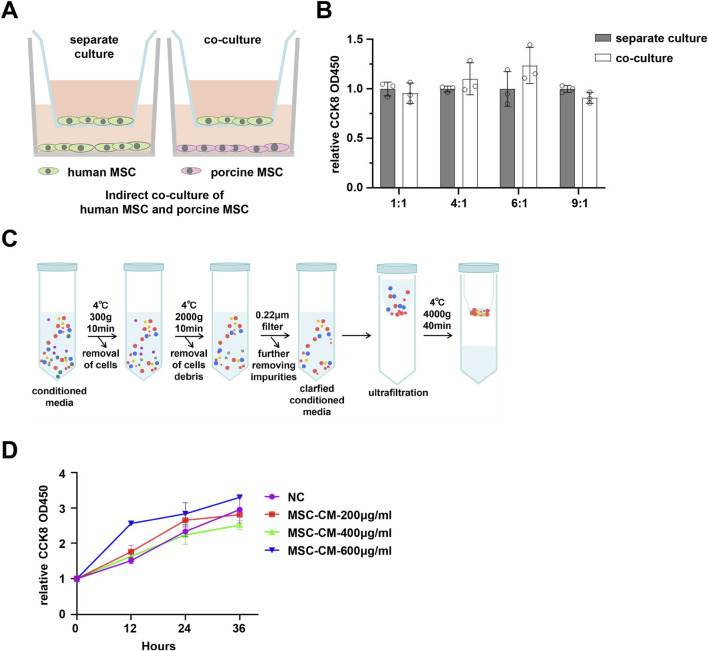
Indirect co-culture of human and porcine MSCs. **(A)** Schematic diagram of the transwell-based indirect co-culture system for human and porcine MSCs. **(B)** The proliferation of hMSCs under transwell indirect co-culture conditions. **(C)** Schematic diagram of the extraction process for conditioned medium. **(D)** The effect of pMSC-derived conditioned medium on hMSC proliferation.

To elucidate the potential mechanisms underlying the inhibition of hMSC proliferation mediated by pMSCs, we employed a transwell non-contact co-culture assay to investigate whether the soluble factors secreted by pMSCs contribute to the suppression of hMSC growth. Notably, the results demonstrated that pMSCs at different proportions in the non-contact co-culture system did not significantly affect hMSC proliferation (Figures 4A,B), suggesting that the inhibitory effect of pMSCs on hMSCs may be attributed to the direct cell-to-cell contact rather than to the secreted soluble factors.

To further validate the role of soluble factors, the conditioned medium (CM) from pMSCs was extracted and purified. The impact of the CM on hMSC proliferation was assessed via CCK-8 assays at varying concentrations and treatment times. The results indicated that concentrations of 200–400 μg/ml of pMSC-CM did not exert a significant effect on hMSC proliferation. Treatment with 600 μg/mL of CM for 12 h induced a slight promotion, which disappeared when the treatment time was extended to 24 h (Figures 4C,D). The modest promotion observed at the 12-h time point for pMSC-CM may attribute to the presence of growth factors, cytokines, and other bioactive components. However, many of these factors have relatively shorter half-lives, complicating the maintenance of their activity over extended periods. Furthermore, subtle variations in the concentrations of active components may occur between different batches of pMSC-CM, contributing to the observed fluctuations in the promotional effect at the 12-h time point across multiple independent replicate experiments. These results suggest that the soluble factors secreted by pMSCs may not be the primary inhibitors of hMSC proliferation.

### 2.4 pMSCs suppress hMSC proliferation by activating the NF-κB pathway

**FIGURE 5:**
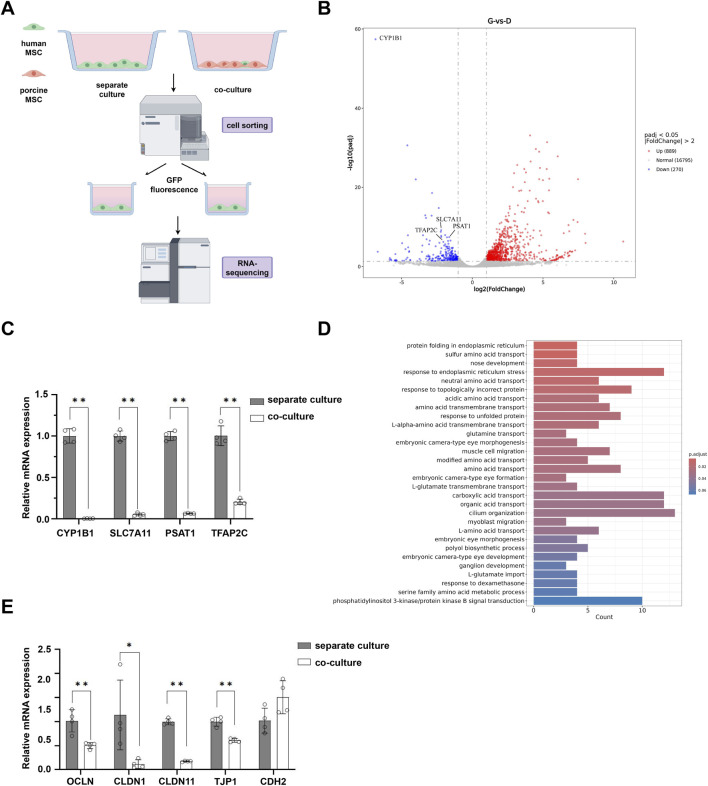
RNA sequencing of hMSCs under direct co-culture conditions. **(A)** Schematic diagram by Figdraw illustrating the experimental procedure that GFP-labeled hMSCs were sorted by flow cytometry from direct co-culture systems, followed by RNA-seq analysis. **(B)** Volcano plot of DEGs in hMSCs. **(C)** qPCR analysis for CYP1B1, SLC7A11, PSAT1 and TFAP2C. **(D)** GO functional enrichment analysis results for DEGs in hMSCs. **(E)** qPCR analysis for OCLN, CLDN1, CLDN11, TJP1 and CDH2. Data are represented as mean ± standard error. *, *P* < 0.05; **, *P* < 0.01.

To elucidate the molecular mechanisms underlying the suppression of hMSC proliferation by pMSCs, we isolated GFP-labelled hMSCs from the co-culture system using cell sorting technology and conducted RNA-seq analysis (Figure 5A). Compared to monoculture, hMSCs in co-culture exhibited significant upregulation of 889 genes and downregulation of 270 genes. Notably, several proliferation-related genes, including *CYP1B1*, *SLC7A11*, *PSAT1*, and *TFAP2C*, were significantly downregulated in the co-culture group, which was further confirmed by qPCR (Figures 5B,C). Gene Ontology (GO) enrichment analysis indicated that the differentially expressed genes were primarily associated with biological processes such as endoplasmic reticulum protein folding, response to misfolded proteins, and amino acid transport, suggesting that co-culture disrupts protein homeostasis and metabolic functions in hMSCs (Figure 5D). Given that direct cell-cell contact could cause an inhibitory effect on proliferation, we next examined the expression alterations of cell junction-related molecules. Notably, significant downregulation of key tight junction molecules, including *OCLN*, *CLDN1*, *CLDN11*, and *TJP1*, was found in co-cultured hMSCs. Among adhesion junction-associated molecules, the baseline expression level of *CDH1* (encoding α-catenin) decreased, whereas *CDH2* (encoding β-catenin) showed no significant difference between groups we tested (Figure 5E).

**FIGURE 6:**
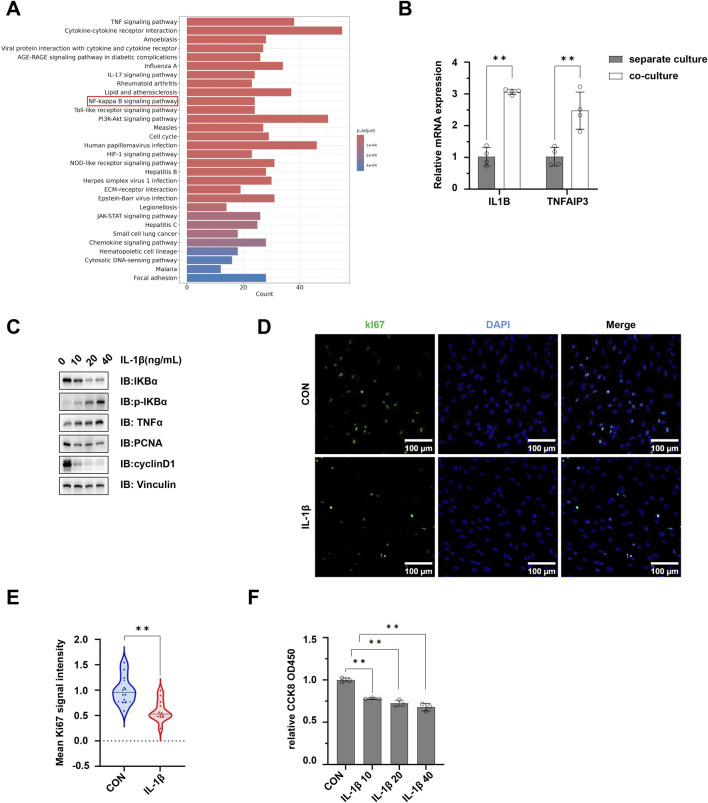
pMSCs suppress hMSC proliferation by activating the NF-κB pathway. **(A)** KEGG pathway enrichment analysis results for DEGs in hMSCs. **(B)** qPCR analysis for IL1B and TNFAIP3. **(C)** Western blot analysis of NF-κB pathway-related proteins IKBα, p-IKBα, TNFα, cyclinD1 and PCNA in hMSCs treated by 10, 20, or 40 ng/mL of IL-1β. **(D)** Immunofluorescence staining of Ki67 protein after IL-1β treatment. **(E)** Statistical analysis of the mean fluorescence intensity of Ki67 protein. **(F)** CCK-8 assay showing the proliferation of hMSCs after IL-1β treatment Data are represented as mean ± standard error. *, *P* < 0.05; **, *P* < 0.01.

Moreover, Kyoto Encyclopedia of Genes and Genomes (KEGG) pathway analysis revealed significant enrichment of signaling pathways associated with cellular competition and proliferation regulation, including NF-κB, PI3K-AKT, and JAK-STAT. Among these, alterations in the NF-κB pathway were particularly pronounced (Figure 6A; Supplementary Figures 2A-C). qPCR results confirmed that the mRNA expression of key NF-κB pathway genes, *IL1B* and *TNFAIP3*, was significantly upregulated in co-cultured hMSCs (Figure 6B). Employing *in vitro* stimulation of hMSCs with interleukin-1β (IL-1β), the activation status of the NF-κB pathway and its role in regulating hMSC proliferation was further investigated. Western blot (WB) analysis demonstrated a decrease in IκBα expression alongside elevated levels of p-IκBα and TNFα, confirming NF-κB pathway activation. Importantly, following NF-κB activation, the expression levels of cyclin D1 and proliferating cell nuclear antigen (PCNA) proteins were significantly downregulated, suggesting an impairment in cellular proliferation capacity (Figure 6C). Further immunofluorescence results indicated a significant reduction in the mean fluorescence intensity of the proliferation marker protein Ki67 in the IL-1β-treated group (Figures 6D,E). Moreover, the CCK-8 assay confirmed that the cell proliferation was markedly suppressed following IL-1β treatment (Figure 6F). These findings collectively suggest that pMSCs impair the proliferative capacity of hMSCs by activating the NF-κB pathway.

## 3 Discussion

Cell competition was first described in *Drosophila* and has been recognized as a widespread phenomenon involved in embryonic development, organ maturation, xenotransplantation, tissue homeostasis, and disease progression. During embryonic development, cell competition serves as an important quality control mechanism to maintain developmental homeostasis by selectively eliminating abnormal cells. In the process of inner cell mass formation, competition driven by differential FGF4 signaling promotes the distinct differentiation of NANOG^+^ epiblast and GATA6^+^ hypoblast cells (Chazaud and Yamanaka, 2016). Studies show that transplanting cells with *Robo4* mutations into bone marrow significantly impairs their niche competitiveness, accompanied by a compensatory increase in *Cxcr4* expression. This suggests that modulating *Robo4* may represent a potential strategy to enhance the competitiveness of hematopoietic stem cells in transplantation settings (Smith-Berdan et al., 2011).

A hallmark of cell competition is the selective elimination of less-fit cells, which often display metabolic and functional impairments, such as aberrant protein synthesis, perturbations in glycolysis, and mitochondrial dysfunction. Studies have shown that RasV12-transformed MDCK cells are eliminated through apical extrusion, a process accompanied by obvious metabolic reprogramming characterized by increased glucose uptake, elevated lactate dehydrogenase expression, heightened lactate secretion, and a complete loss of mitochondrial activity. Importantly, the inhibition of pyruvate dehydrogenase kinase four activity can rescue these cells from extrusion (Kon et al., 2017). Metabolic differences between adjacent cells are key factors determining competitive fitness. For example, cells with elevated *Myc* expression undergo a glycolytic shift, significantly enhancing their competitive ability and proliferative capacity via a p53 dependent pathway (de la Cova et al., 2014). In mice carrying a mutation in the ribosomal protein Rpl24, mutant cells are gradually lost during chimera formation with wild-type cells, indicating that ribosomal dysfunction confers a competitive disadvantage (Edward R et al., 2004).

Signaling pathways such as NF-κB, PI3K-AKT, JAK-STAT, Hippo, Notch, and mTOR play crucial roles in regulating the outcomes of cellular competition. Specifically, the NF-κB and JAK-STAT pathways mediate the elimination of disadvantaged cells. In *Myc*-overexpression-induced cell competition, mutations in the NF-κB homolog Rel or its key regulator Dredd impede the apoptotic clearance of these disadvantaged cells (Meyer et al., 2014). Furthermore, cells exhibiting elevated STAT activity can eliminate disadvantaged cells through hid-dependent mechanisms (Rodrigues et al., 2012). The PI3K-AKT pathway positively regulates cell survival and competitive advantage, and its synergistic activation with *Myc* overexpression significantly enhances the acquisition of winner status (Sander et al., 2012). Notch signaling demonstrates environment-dependent regulation in mammalian systems. In the esophageal epithelium, Notch-inhibited cells achieve clonal proliferation, potentially by promoting the differentiation and clearance of surrounding wild-type cells (Alcolea et al., 2014). Inhibition of the Hippo and mTOR pathways typically renders cells susceptible to elimination. During embryonic ectoderm formation, cells with reduced expression of TEAD/YAP, downstream effectors of the Hippo pathway, are eliminated via apoptosis (Hashimoto and Sasaki, 2019). Similarly, cells with suppressed mTOR signaling are eliminated when coexisting with wild-type cells, whereas abnormal activation of mTOR signaling under stress may induce apoptosis through Bax-mediated pathways (Javedan et al., 2016; Bowling et al., 2018).

Overcoming interspecies cellular competition barriers is a fundamental challenge in enhancing the efficiency of xenotransplantation of human-derived cells. In interspecies co-culture systems, competitive interactions between host cells and transplanted cells frequently suppress the survival and proliferation of human cells, significantly constraining chimerism efficiency. Wang et al. found that activation of the NF-κB signaling pathway in human PSCs led to their competitive elimination in a human-mouse PSCs co-culture system. Consistently, inhibiting p65 or MYD88 in human cells effectively counteracted this competition, substantially improving human cell survival and chimerism in mouse early embryos (Zheng et al., 2021). Subsequent research by the same team revealed interspecies RNA exchange within human-mouse PSCs co-culture systems, activating the RNA innate immune pathway in mouse cells and mediating the competitive clearance of human cells. Knocking out key proteins RIG-I or MAVS in this pathway significantly weakened the competitive advantage of mouse cells, thereby substantially improving human cell survival rates and embryonic chimerism rates without modifying human cells (Hu et al., 2025).

The low survival rate of human-derived cells in pigs remains a significant bottleneck that restricts the clinical application of xenotransplantation. However, the impact of porcine cells on human cell growth has rarely been investigated. Given the absence of established porcine PSCs with high chimeric capability, this study developed an *in vitro* direct co-culture model of hMSCs and pMSCs to investigate the effect of interspecies cellular competition. Our results demonstrated that, compared to hMSCs cultured alone, the proliferation of hMSCs in the co-culture system was significantly suppressed. Further adjustments to the seeding ratios revealed that as the proportion of pMSCs increased, the inhibitory effect on hMSC proliferation correspondingly intensified. Furthermore, transwell indirect co-culture and CM treatment experiments confirmed that the inhibition of hMSC proliferation by pMSCs is independent of paracrine factors but closely associated with direct cell-cell contact. To further investigate the underlying molecular mechanisms, we performed RNA sequencing analysis and found that direct hMSC–pMSC contact significantly downregulated the expression of several proliferation-related genes in hMSCs, including *CYP1B1*, *SLC7A11*, *TFAP2C*, and *PSAT1* (Kang et al., 2016; Al-Dhfyan et al., 2017; Fantone et al., 2024; Liu et al., 2025). Previous studies have demonstrated that tight junctions and adherens junctions significantly influence the outcomes of cellular competition (Skamrahl et al., 2021). Our analysis on the expression patterns of genes related to cell junctions revealed that the mRNA levels of key tight junction molecules, *OCLN*, *CLDN1*, *CLDN11*, and *TJP1*, were significantly downregulated in the co-culture system, whereas the expression of *CDH2* in adherens junctions remained unchanged. This suggests that cell contact exerts its effects by influencing the structure or function of tight junctions.

In the present study, GO enrichment analysis revealed that differentially expressed genes were primarily associated with endoplasmic reticulum protein folding, responses to misfolded proteins, and amino acid transport. This suggests that interspecies cell competition between human and porcine cells may disrupt protein processing homeostasis and metabolic functions in hMSCs, thereby affecting their survival and proliferative capacity. KEGG pathway enrichment analysis revealed significant activation of pathways closely linked to cellular competition and proliferation regulation, including NF-κB, PI3K-AKT, and JAK-STAT, in co-cultured hMSCs, with the enrichment of the NF-κB pathway being the most pronounced. Excessive activation of the NF-κB pathway may represent a key mechanism underlying the competitive disadvantage of hMSCs in co-culture conditions. Consequently, modulating the activity of this pathway holds promise for mitigating the competitive disadvantage of hMSCs, thereby enhancing their survival rates within complex microenvironments such as allogeneic transplantation.

## 4 Conclusion

In a direct co-culture system involving human and porcine MSCs, the presence of pMSCs inhibits the proliferation of hMSCs. RNA sequencing results indicate that direct co-culture significantly downregulates the expression of proliferation-related genes in hMSCs, disrupting endoplasmic reticulum function and amino acid transport processes. Further investigations revealed that pMSCs impair the proliferative capacity of hMSCs partially by activating the NF-κB pathway in the latter.

## 5 Materials and methods

### 5.1 Cell culture

Human fetal bone marrow MSCs were purchased from Cyagen Biosciences (HUXMF-01001, China). hMSCs and pMSCs were cultured in low-glucose Dulbecco’s Modified Eagle’s Medium (DMEM) (Gibco, USA) supplemented with 10% fetal bovine serum (OriCell, China), 1% non-essential amino acids (Gibco, USA), 1% GlutaMAX (Gibco, USA), and 1% penicillin-streptomycin (Transgen, China) at 95% humidity and 5% CO_2_, with temperatures set at either 37 °C or 39 °C. HEK293FT cells were maintained in DMEM (Gibco, USA) supplemented with 10% fetal bovine serum and 1% penicillin-streptomycin.

### 5.2 Extraction of pMSCs

Healthy piglets within 7 days postnatally were selected. Under aseptic conditions, the femurs and tibiae were dissected, disinfected twice with 75% ethanol, and rinsed three times with phosphate-buffered saline (PBS) containing penicillin-streptomycin. Bone marrow was flushed out using complete medium and cultured at 37 °C with 5% CO_2_ under 95% humidity. Half of the medium was replaced every 12 h. After 72 h, the culture dish was washed twice with PBS to remove non-adherent cells, and complete medium was replenished for continued culture. Thereafter, the medium was changed every 3 days. Upon reaching 80%–90% confluence, the cells were either cryopreserved or passaged for subsequent experiments.

### 5.3 Expression of pMSCs surface markers

pMSCs (1 × 10^6^) were washed with FACS buffer and resuspended in 100 μL of FACS buffer. The cells were incubated at 4 °C for 30 min in the dark with fluorochrome-conjugated primary antibodies: anti-CD90 APC (559869, BD), anti-CD44 BV785 (103041, BD), anti-CD45 BV480 (566115, BD), anti-CD14 BV650 (563420, BD), and anti-CD34 FITC (555821, BD). After washing, the cells were analyzed using A5 flow cytometry, and the data were processed using FlowJo software.

### 5.4 Osteogenic differentiation

pMSCs were seeded into a 12-well plate at a density of 2 × 10^4^ cells per well. Once the cell density reached 70%, the medium was replaced with osteogenic induction medium (OriCell, China), which was changed every 3 days during the osteogenic differentiation process. On day 9, when calcium nodule formation was observed, the cells were fixed with 4% paraformaldehyde (PFA) for 30 min. Subsequently, the cells were stained with alizarin red for 5–10 min. The results were then observed and photographed under microscope.

### 5.5 Lipidogenic differentiation

Cells were seeded into a 12-well plate at a density of 3 × 10^4^ cells per well. Once the cell density reached 100%, the medium was replaced with adipogenic induction medium A (OriCell, China). After 3 days of culture, medium A was substituted with induction medium B, and the two media were alternated thereafter. Approximately 2 weeks later, when lipid droplet formation was observed, the cells were fixed with 4% PFA for 30 min. Subsequently, the cells were stained with Oil Red O for 30 min. The results were then observed and photographed under a microscope.

### 5.6 Chondrogenic differentiation

pMSCs were cultured at a density of 4 × 10^5^ cells in 15 mL tubes and centrifuged at 250 *g* for 5 min. The cell pellets were resuspended in 0.5 mL of chondrogenic media (OriCell, China) and subjected to a second centrifugation at 150 *g* for 5 min. The chondrogenic media was replaced every 2 days until chondrospheres were observed. Subsequently, the chondrospheres were fixed in 4% PFA for 30 min. Following fixation, the chondrospheres were embedded in optimal cutting temperature compound and stored at −80 °C. Continuous frozen sections were prepared, and Alcian blue staining was performed. The results were then observed and photographed under a microscope.

### 5.7 Cell proliferation assays

Cell proliferation assays were conducted using a CCK-8 reagent (Beyotime, China). Cells were seeded in 96-well plates at a density of 1 × 10^3^ cells per well in 100 μL of complete medium. At the indicated time points, 10 μL of CCK-8 reagent was added to each well and incubated at 37 °C for 2 h. The absorbance was measured at 450 nm. All samples were tested in three independent experiments.

### 5.8 Generation of GFP-hMSCs

The pLenti-CMV-GFP cloning vector (Addgene 17448), psPAX2, and pMD2.G were co-transfected into HEK293FT cells using Lipofectamine 2000 reagent (Invitrogen, USA) according to the manufacturer’s instructions. The culture supernatant containing lentivirus was collected and concentrated using ultrafiltration centrifuge tubes (Millipore, USA). MSCs were seeded in a 12-well plate at a density of 3 × 10^4^ cells per well and cultured until reaching 30%–40% confluence. The cells were infected with the concentrated lentivirus and 5 μg/mL Polybrene (Sigma, USA) to enhance transduction efficiency. After 12 h of infection, the virus-containing medium was removed, and fresh complete medium was added for continued cultivation. About 7 days later, GFP-positive cells were sorted using a BD flow cytometer (BD, USA) after digestion with 0.25% trypsin and filtration through a 70 μm sterile cell strainer. The sorted cells were cultured for 1 week to establish stable cell lines. The transduction efficiency of the lentivirus was confirmed through qPCR and WB analysis.

### 5.9 Direct co-culture of human and porcine MSCs

The experiment included five groups: hMSCs cultured alone, and hMSCs co-cultured with pMSCs at ratios of 1:1, 1:4, 1:6, and 1:9. The initial total cell number was kept postnatally across all groups by adjusting the seeding density of each cell type. On days 4 and 8 after seeding, the cells were digested and collected. The total cell number was obtained using a cell counter, and the proportion of GFP-positive hMSCs was determined by flow cytometry. The absolute number of hMSCs in each group was calculated by multiplying the total cell count by the percentage of GFP-positive cells. Data were processed using the formula: normalized growth curve = log_e_(final cell number/initial cell number)/log_e_2, and the normalized growth curve of hMSCs was plotted accordingly.

### 5.10 Immunofluorescence staining (IF)

The indicated cells (1 × 10^4^) were seeded on glass slides in 24-well plates. The cells were washed with PBS and fixed in 4% PFA for 20 min at room temperature. Fixed cells were permeabilized with 1% Triton X-100 for 10 min and blocked with 1% bovine serum albumin supplemented with 22.52 mg/mL glycine for 30 min at room temperature. Subsequently, the cells were incubated overnight at 4 °C with primary antibodies: c-caspase3 (Cell Signaling Technology, USA) or Ki67 (Abcam, USA). The cells were then washed three times with PBST (PBS +0.1% Tween 20) and incubated at room temperature for 1 h with secondary antibodies: goat anti-rabbit IgG H&L (Alexa Fluor® 555) (Abcam, USA) or goat anti-rabbit IgG H&L (Alexa Fluor® 488) (Abcam, USA). The cell nuclei were stained with DAPI (Beyotime, China) for 15 min at room temperature in the dark. Finally, images were captured using a Zeiss LSM880 confocal microscope.

### 5.11 Transwell indirect co-culture of human and porcine MSCs

Human and porcine MSCs were indirectly co-cultured in a Transwell system with 0.4 μm pores. The upper chamber was seeded with hMSCs (1 × 10^4^), while the lower chamber contained either hMSCs (separate culture) or pMSCs (co-culture) at varying seeding ratios. After 4 days of culture, the proliferation of hMSCs in the upper chamber was assessed using the CCK-8 kit.

### 5.12 Preparation of conditioned medium from pMSCs

The pMSCs with 80%–90% confluence were washed three times with PBS and cultured in serum-free medium for 24–48 h. The supernatant was collected and centrifuged at 300 *g* for 10 min at 4 °C to remove residual cells, followed by a second centrifugation at 2000 *g* for 10 min at 4 °C to pellet cellular debris. The solution was then filtered through a 0.22 μm membrane and concentrated using a 3 kDa Millipore ultrafiltration device by centrifugation at 4,000 *g* for 40 min at 4 °C. The protein concentration of the CM was quantified using the Pierce™ BCA Protein Detection Kit (Thermo, USA) and stored at −80 °C for future use.

### 5.13 RNA sequencing

A total of 16 × 10^4^ GFP-labeled hMSCs (single-culture group) or 2.28 × 10^4^ GFP-labeled hMSCs combined with 13.72 × 10^4^ pMSCs (co-culture group) were seeded into 10 cm culture dishes, with three replicates for each group. Once the cells reached confluence, they were digested, centrifuged at 1,000 rpm for 5 min, and resuspended in 800 μL of FACS buffer. The suspension was then filtered through a 70 μm sterile cell strainer. GFP-expressing hMSCs (5 × 10^4^ cells) were isolated using cell sorting, washed with PBS, and centrifuged again at 1,000 rpm for 5 min. The resulting cell pellet was resuspended in 400 μL of TRIzol and stored at −80 °C.

Total RNA was extracted from MSCs using TRIzol reagent (Invitrogen, USA) in accordance with the manufacturer’s protocol. For single library preparation, the total RNA requirement was set at ≥1 μg, with a concentration of ≥35 ng/μL, an OD260/280 ratio of ≥1.8, and an OD260/230 ratio of ≥1.0. RNA concentration and purity were assessed using the ND-2000 spectrophotometer (NanoDrop, USA). RNA purification, reverse transcription, library preparation, and sequencing were performed following the operational guidelines (Illumina, USA). Sequence libraries underwent 2 × 150 bp paired-end sequencing on the Illumina NovaSeq 6,000 platform, facilitated by Cosmos Wisdom Biotech Co., Ltd. (Hangzhou, China). Raw data were filtered using fastp software to remove adapter-containing reads, trim terminal N bases, and discard low-quality reads (reads where bases with Q_phred_ ≤ 20 constitute over 50% of the total read length). Alignment against the human reference genome (*Homo sapiens* GRCh38 release-109) was conducted using Hisat2 software. Based on the alignment results, differentially expressed genes (DEGs) were analyzed using the DESeq2 R package. Genes were identified as differentially expressed if they met the criteria of a P-value ≤0.05 and an absolute fold change ≥2. GO and KEGG enrichment analyses of DEGs were conducted using the cluster Profiler R package, with enrichment significance assessed using a corrected P-value ≤ 0.05 threshold. Finally, ggplot2 was utilized to visualize the enrichment results.

### 5.14 Quantitative real-time PCR

Total RNA was extracted from the MSCs using TRIzol (Invitrogen, USA). The purity and concentration of the RNA were determined using a NanoDrop ND-2000 spectrophotometer (Thermo, USA), with an A260/A280 ratio of 1.8–2.0 considered acceptable. The RNA was reverse-transcribed into cDNA using RevertAid Master Mix (Thermo, USA). The validated or designed primer sets utilized are listed in Table 1 qPCR was performed on a StepOnePlus™ Real-Time PCR System (Applied Biosystems, USA). Mean cycle threshold values from triplicate measurements were used to calculate gene expression using the 2^−ΔΔCt^ method, normalizing to GAPDH as the internal control.

**TABLE 1:** The primers used in the research.

Gene	Primer sequence
CYP1B1 forward primer	5′-AAG​TTC​TTG​AGG​CAC​TGC​GAA-3′
CYP1B1 reverse primer	5′-GGC​CGG​TAC​GTT​CTC​CAA​AT-3′
SLC7A11 forward primer	5′-TCT​CCA​AAG​GAG​GTT​ACC​TGC-3′
SLC7A11 reverse primer	5′-AGA​CTC​CCC​TCA​GTA​AAG​TGA​C-3′
PSAT1 forward primer	5′-TGC​CGC​ACT​CAG​TGT​TGT​TAG-3′
PSAT1 reverse primer	5′-GCA​ATT​CCC​GCA​CAA​GAT​TCT-3′
TFAP2C forward primer	5′-TCA​GTC​CCT​GGA​AGA​TTG​TCG-3′
TFAP2C reverse primer	5′-CCA​GTA​ACG​AGG​CAT​TTA​AGC​A-3′
OCLN forward primer	5′-GAC​TTC​AGG​CAG​CCT​CGT​TAC-3′
OCLN reverse primer	5′-GCC​AGT​TGT​GTA​GTC​TGT​CTC​A-3′
CLDN1 forward primer	5′-CCT​CCT​GGG​AGT​GAT​AGC​AAT-3′
CLDN1 reverse primer	5′-GGC​AAC​TAA​AAT​AGC​CAG​ACC​T-3′
CLDN11 forward primer	5′-CAT​TTT​ACT​GCT​GCT​GAC​TGT​T-3′
CLDN11 reverse primer	5′-CAG​AAT​GAG​CAA​AAC​ACC​AGC-3′
TJP1 forward primer	5′-CAA​CAT​ACA​GTG​ACG​CTT​CAC​A-3′
TJP1 reverse primer	5′-CAC​TAT​TGA​CGT​TTC​CCC​ACT​C-3′
CDH1 forward primer	5′-CGA​GAG​CTA​CAC​GTT​CAC​GG-3′
CDH1 reverse primer	5′-GGG​TGT​CGA​GGG​AAA​AAT​AGG-3′
CDH2 forward primer	5′-AGC​CAA​CCT​TAA​CTG​AGG​AGT-3′
CDH2 reverse primer	5′-GGC​AAG​TTG​ATT​GGA​GGG​ATG-3′
IL1B forward primer	5′-ATG​ATG​GCT​TAT​TAC​AGT​GGC​AA-3′
IL1B reverse primer	5′-GTC​GGA​GAT​TCG​TAG​CTG​GA-3′
TNFAIP3 forward primer	5′-TCC​TCA​GGC​TTT​GTA​TTT​GAG​C-3′
TNFAIP3 reverse primer	5′-TGT​GTA​TCG​GTG​CAT​GGT​TTT​A-3′
GAPDH forward primer	5′-GGA​GCG​AGA​TCC​CTC​CAA​AAT-3′
GAPDH reverse primer	5′-GGC​TGT​TGT​CAT​ACT​TCT​CAT​GG-3′

### 5.15 IL-1β treatment in hMSCs

When hMSCs reached 70% confluence, 10, 20, or 40 ng/mL of IL-1β (Peprotech, USA) was added into the culture medium to activate the NF-κB pathway. The cells were harvested for subsequent Western blot analyses at the indicated time points.

### 5.16 Western blot (WB)

Cells were lysed in EBC buffer (50 mM Tris pH 7.5, 120 mM NaCl, 0.5% NP-40), supplemented with protease inhibitors (Complete Mini, Roche) and phosphatase inhibitors (phosphatase inhibitor cocktail sets A and B, Biomake). Cell lysates were centrifuged at 12,000 × g for 15 min at 4 °C, and the supernatant was collected. Protein concentrations were determined using the Pierce™ BCA Protein Detection Kit (Thermo, USA). Approximately 30 μg of protein was analyzed via 10% SDS-polyacrylamide gel electrophoresis. The resolved proteins were transferred onto polyvinylidene difluoride membranes, which were blocked with 5% skim milk for 1 h at room temperature. The membranes were incubated for 12 h with primary antibodies: anti-TNFα (Proteintech, China), anti-p-IKBα (Selleck, USA), anti-cyclinD1 and anti-PCNA (Immunoway, China) and anti-IKBα (Abcam, USA) (Su et al., 2014; Aierken et al., 2022; Qian et al., 2024; Zhao et al., 2025; Zhou et al., 2025). To avoid issues of target band overlap and interference from non-specific bands, we selected the anti-Vinculin antibody (Sigma, USA) as our internal control (Wenzel et al., 2024). The membranes were then washed three times with TBST (20 mM Tris-HCl pH 7.6, 150 mM NaCl, 0.1% Tween 20) and incubated with the corresponding secondary antibodies: goat anti-rabbit IgG H&L or goat anti-mouse IgG H&L (Abcam, USA) at room temperature for 1 h. Signals were detected using Immobilon Western Chemiluminescent HRP Substrate (Thermo, USA) with an image analyzer.

### 5.17 Statistical analysis

All experiments were performed with at least three independent biological replicates. Values were represented as mean ± standard error of the mean (SEM). Data analysis was performed GraphPad Prism 9 statistical software. Comparisons between two groups were analyzed using *t*-test. *P* < 0.05 was considered statistically significant, and *P* < 0.01 was considered highly statistically significant.

## Data Availability

RNA-seq data have been deposited in the China National Center for Bioinformation - National Genomics Data Center (CNCB-NGDC) under accession number PRJCA048099.
