# Supplementary material for: Porcine cells restrict human cell proliferation via cellular competition in a human-porcine mesenchymal stem cells co-culture model
Source: Front Cell Dev Biol. 2026 Feb 25;14:1750289. doi: 10.3389/fcell.2026.1750289 (PMC12976001; doi:10.3389/fcell.2026.1750289)
Supplement: Supplementary file 1 [file DataSheet1.docx]

**
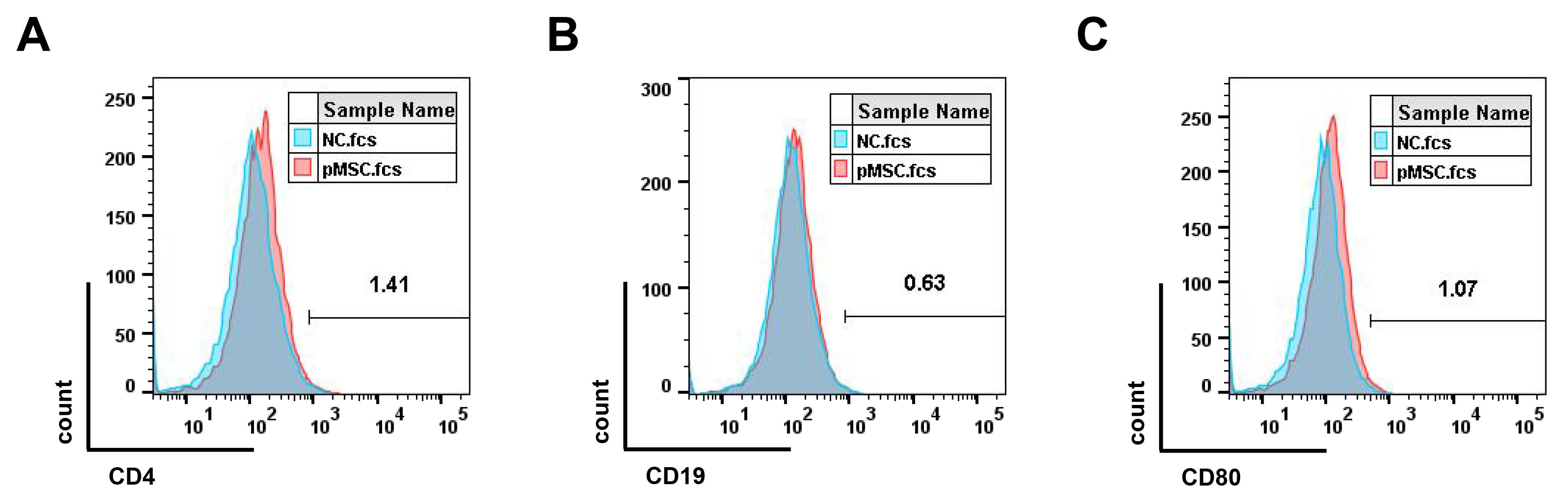
**

**Supplementary Figure 1 Flow cytometric analysis of surface marker on pMSCs.**

(A) Flow cytometric analysis of CD4 surface marker expression in pMSCs.

(B) Flow cytometric analysis of CD19 surface marker expression in pMSCs.

(C) Flow cytometric analysis of CD80 surface marker expression in pMSCs.


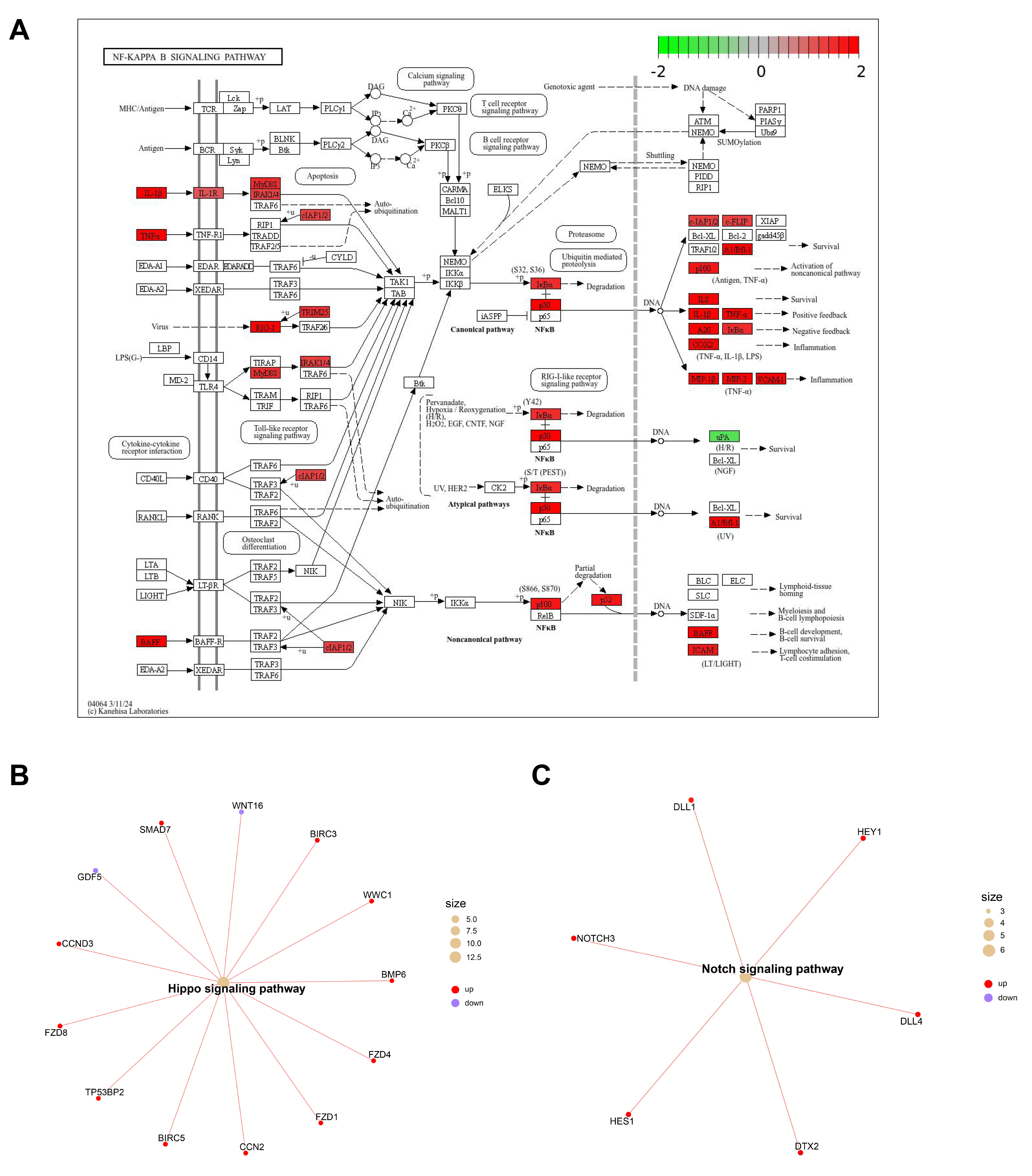


**Supplementary Figure 2** **Enrichment and regulatory networks of NF-κB, Hippo, and Notch signaling pathways in RNA sequencing**

(A) Regulatory network diagram of the NF-κB signalling pathway.

(B) Enrichment network diagram of the Hippo signalling pathway.

(C) Enrichment network diagram of the Notch signalling pathway.
